# Genome-wide RNA-binding analysis of the trypanosome U1 snRNP proteins U1C and U1-70K reveals *cis*/*trans*-spliceosomal network

**DOI:** 10.1093/nar/gku286

**Published:** 2014-04-19

**Authors:** Christian Preußer, Oliver Rossbach, Lee-Hsueh Hung, Dan Li, Albrecht Bindereif

**Affiliations:** Institute of Biochemistry, Justus Liebig University of Giessen, D-35392 Giessen, Germany

## Abstract

*Trans-*splicing in trypanosomes adds a 39-nucleotide mini-exon from the spliced leader (SL) RNA to the 5′ end of each protein-coding sequence. On the other hand, *cis-*splicing of the few intron-containing genes requires the U1 small nuclear ribonucleoprotein (snRNP) particle. To search for potential new functions of the U1 snRNP in *Trypanosoma brucei*, we applied genome-wide individual-nucleotide resolution crosslinking-immunoprecipitation (iCLIP), focusing on the U1 snRNP-specific proteins U1C and U1-70K. Surprisingly, U1C and U1-70K interact not only with the U1, but also with U6 and SL RNAs. In addition, mapping of crosslinks to the *cis*-spliced *PAP* [poly(A) polymerase] pre-mRNA indicate an active role of these proteins in 5′ splice site recognition. In sum, our results demonstrate that the iCLIP approach provides insight into stable and transient RNA–protein contacts within the spliceosomal network. We propose that the U1 snRNP may represent an evolutionary link between the *cis-* and *trans*-splicing machineries, playing a dual role in 5′ splice site recognition on the *trans*-spliceosomal SL RNP as well as on pre-mRNA *cis*-introns.

## INTRODUCTION

Pre-mRNA splicing, an essential step between transcription and translation of most eukaryotic mRNAs, is catalyzed by a macromolecular complex termed the spliceosome. Consisting of small nuclear ribonucleoproteins (snRNPs) and other protein components, the spliceosome assembles in a stepwise manner on the precursor mRNAs. In *Trypanosoma brucei*, the expression of protein-coding genes, in particular the mRNA-processing stages, differs in several respects from other eukaryotes: protein-coding genes are organized in long polycistronic transcription units, and mRNA maturation requires coupled *trans-*splicing and polyadenylation steps. During *trans-*splicing, the spliced leader RNA (SL RNA), which is a constituent of the SL RNP, adds the 39-nucleotide mini-exon from its 5′ end to every protein-coding pre-mRNA, thereby generating SL-capped mRNAs. In addition to the SL RNP, the U2, U4/U6 and U5 snRNPs are essential *trans*-splicing factors (for review, see (1)).

As confirmed by recent genome-wide studies (2,3), only two genes with intronic sequences were identified in *T. brucei*, coding for *PAP* [poly(A) polymerase; Tb927.3.3160] and a putative, ATP-dependent DEAD/H RNA helicase (Tb927.8.1510). Both contain a single intron, which is removed by *cis-*splicing. Although these are likely the only two *cis*-introns in *T. brucei*, this explains the existence of a U1 snRNP in trypanosomes: *cis*-splicing requires the recognition of the 5′ splice sites through base-pairing between the U1 snRNA and the 5′ splice site on the pre-mRNA (4). The trypanosome U1 snRNP is unusual in several aspects: its three specific protein components, U1-70K, U1C and U1A, are only distantly related to their known counterparts from other eukaryotes; in addition, U1-24K was characterized as a trypanosomatid-specific U1 snRNP protein, which is stably integrated into the U1 snRNP by protein–protein interactions (5,6). Interestingly, the trypanosome U1 snRNA with 75 nucleotides represents one of the smallest known snRNAs, and lacks a stem-loop II element, which in other orthologs contains the well-characterized U1A binding site.

Does the U1 snRNP function in trypanosomes only in *cis*-splicing of two introns, or are there additional functions beyond splicing? In other systems, in particular the mammalian system, there are several lines of evidence for splicing-independent roles of the U1 snRNP and its protein components, which seem plausible based on the relatively high abundance of the U1 snRNP:

First, the U1 snRNP was found to be recruited to intronless genes (7). Second, the U1-specific protein U1A inhibits polyadenylation of its own and other pre-mRNAs by interacting with polyadenylation factors (8,9). Third, a genome-wide study demonstrated that the U1 snRNP can protect pre-mRNAs from premature cleavage and polyadenylation by binding to cryptic 5′ splice sites (10,11). Fourth, the U1 snRNP-specific protein U1C plays a peculiar role beyond constitutive splicing. Specifically, the efficient assembly of the U1 snRNP on the 5′ splice site requires U1C, by stabilizing base-pairing between the 5′ end of the U1 snRNA and the 5′ splice site region (12–16), a role that appears to be U1 snRNA-independent (17). Moreover, a recent global RNA-Seq study revealed a novel role of U1C during alternative splicing, primarily during 5′ splice site recognition (18).

In line with these studies, tandem-affinity purification of U1A in trypanosomes identified a large collection of co¬purifying factors, among them the polyadenylation factor CPSF73, suggesting a role of U1A in coupling 3′-processing and splicing (19).

Recently, four independent RNA-Seq analyses provided evidence that alternative *trans-*splicing and polyadenylation are more common in trypanosomes than previously thought, raising the question how these mRNA-processing steps in trypanosomes are regulated (3,20–22).

To obtain more insight into known and novel regulatory functions of the U1 snRNP in trypanosomes, we have adapted the individual-nucleotide resolution crosslinking-immunoprecipitation (iCLIP) approach (23), combined with deep-sequencing, to the trypanosome system. Genome-wide mapping of the crosslinks generated a comprehensive map of U1C- and U1-70K RNA-interaction sites: not only the U1 snRNA, but, surprisingly, also the SL RNA and the U6 snRNA are prominent targets of U1C and U1-70K. In addition, mapping of U1C crosslinks to the *cis*-spliced *PAP* pre-mRNA indicate an active role of these U1 snRNP proteins in 5′ splice site recognition. Taken together, our results demonstrate that the iCLIP approach allows insight into stable and transient RNA–protein contacts within the spliceosomal network. We propose that the U1 snRNP may represent an evolutionary link between the *cis*- and *trans*-splicing machineries, playing a dual role in 5′ splice site recognition on the SL RNP as well as on pre-mRNA *cis*-introns.

## MATERIALS AND METHODS

### Cell culture and extract preparation

For the generation of cell lines expressing PTP-tagged U1C and U1-70K, the pC-PTP-Neo vector including the ORF of TbU1-70K (nts 88–831) was used (24). For U1C, the ORF (nts 13–582) of *T*. *brucei* U1C was PCR-amplified and inserted in-frame into the pC-PTP-NEO vector upstream of the PTP tag sequence, using ApaI and NotI restriction sites. For genomic integration, 10 μg of linearized pC-PTP-constructs were transfected into procyclic *T*. *brucei* 427 and cloned by limiting dilution in the presence of G418 (40 μg/ml Geneticin; Gibco-BRL).

Cell culture of *T. brucei* 427 and 29-13, was described previously (24,25). Cell lysates were prepared in extraction buffer (500 mM KCl, 20 mM Tris–Cl, pH 7.7, 3 mM MgCl_2_, 0.5 mM DTT), containing a Complete Mini, EDTA-free protease inhibitor cocktail tablet (Roche), using a Dounce homogenizer followed by sonication. Cell lysates were supplemented with 0.1% Tween-20, and centrifuged twice at 14 000 rpm for 15 min to remove aggregates.

For starvation experiments, cells (logarithmic phase) were collected, washed twice in phosphate-buffered saline (PBS), resuspended in the original volume of PBS, incubated at 27°C for 90 min, and then returned to pre-warmed SDM-79 and incubated at 27°C.

### Immunofluorescence

The cellular distribution of U1C-PTP by indirect immunofluorescence was analyzed as described (26).

### iCLIP-Seq

Three (U1C-PTP) and two (U1-70K) biological replicates of iCLIP experiments were performed for each of the stable cell lines. *Trypanosoma brucei* 427 wild-type (WT) cells served as a negative control in each replicate. The iCLIP procedure was performed as described by König *et al.* (23), with minor modifications (see below), and combined with tandem-affinity purification (24). 5 × 10^8^ procylic *T. brucei* cells were irradiated with UV-C light (3 × 300 mJ/cm^2^). Lysates were prepared in 4 ml extraction buffer (500 mM KCl, 20 mM Tris–Cl, pH 7.7, 3 mM MgCl_2_, 0.5 mM DTT) using a Dounce homogenizer (25 strokes with a type B pestle) followed by sonication. Extracts were cleared by centrifugation at 14 000 rpm for 30 min and subsequently, 1 ml of cleared extract was subjected to combined DNase treatment (TURBO™ DNase, Ambion, at a final concentration of 4 U/ml), and limited RNase digestion (RNase I, Ambion, at a final concentration of 0.01 U/ml), for 3 min at 37°C. Lysates were centrifuged at 14 000 rpm for 30 min to remove aggregates. The iCLIP library preparation steps were exactly performed as described by König *et al.* (23), except of the tandem-affinity purification steps. In brief, U1C- or U1-70K RNA–protein complexes were purified by applying the first step of tandem-affinity purification (IgG Sepharose 6 Fast Flow, GE Healthcare), followed by phosphatase treatment, ligation of an RNA adapter at the 3′ ends of the RNA tags (T4 RNA ligase; Thermo Scientific) and radiolabeling using polynucleotide kinase treatment to allow visualization of covalent RNA–protein complexes. By *tobacco-etch-virus* (TEV) protease bound material was released from the beads, followed by the second affinity step (anti-protein C immunoaffinity purification). Purified RNA–protein complexes were subjected to sodium dodecyl sulfate-polyacrylamide gel electrophoresis (SDS-PAGE), followed by electro-blotting. Complexes were then recovered by proteinase K treatment. cDNA was generated by reverse transcription (Superscript III; Life Technologies), using oligonucleotides, which introduce a 5′-barcode as well as a BamHI restriction site. cDNAs obtained were size-fractionated by denaturing polyacrylamide gel electrophoresis, circularized (Circligase II, Epicentre), annealed to an oligonucleotide complementary to the BamHI restriction site, and cut between the two adapter regions by BamHI. Linearized molecules were then PCR amplified (27–32 cycles), using primers with sequencing adapters.

U1C iCLIP libraries were sequenced either on an Illumina GAIIx (U1C_1, U1C_2; 105-bp single-end reads) and or on an Ion Torrent PGM (U1C_3; single-end reads with diverse lengths); U1-70K iCLIP libraries were sequenced on the Illumina MiSeq (U1-70K_1, U1-70K_2; 50-bp single-end reads).

The sample- and random-barcode sequences were removed from the 5′ end, followed by linker sequence trimming at the 3′ end. Trimmed sequence reads with a minimum length of 15 bp were aligned to the *T. brucei* 427 genomic sequence (Tbrucei427Genomic_TriTrypDB-4.2.fasta; see http://tritrypdb.org). The gene annotation file (Tbrucei427_TriTrypDB-3.3.gff; see http://tritrypdb.org) was used for functional analysis. Reads mapped to tRNAs and rRNAs were excluded, and only uniquely mapped reads were selected as iCLIP tags for crosslink-site analysis (for details, see (23)). The raw data containing all sequence reads as well as the processed data containing all barcode-filtered tag counts of crosslink sites in the Tb427 genome and in SL, U1 and U6 RNAs were deposited (NCBI GEO database: GSE43848).

### RNA interference (RNAi) silencing of U1C expression and real-time RT-PCR

The RNAi construct pLEW100-U1C was made, using the stem-loop vector pLEW100 according to an established cloning strategy (27). The resulting construct was linearized with SacII, and 10 μg were transfected into *T. brucei* 29-13 cells by electroporation. Transformants were cloned by limiting dilution in the presence of G418 (15 μg/ml), hygromycin (50 μg/ml) and phleomycin (2.5 μg/ml). RNAi was induced by the addition of 1 μg/ml of doxycycline. Cells were counted every day and diluted to 2 × 10^6^ cells/ml. Semiquantitative as well as quantitative real-time RT-PCR were performed as described (26).

### RNA analysis

RNA extraction, northern blot analysis and silver staining were performed as described (26). For protein–RNA crosslinking, formaldehyde (at a final concentration of 1%) was added to 5 × 10^7^ cells in 20 ml SDM-79 medium, incubated for 20 min, and fixation was quenched by the addition of glycine (125 mM) for 5 min, while rotating at room temperature. Cells were washed in 1× PBS, followed by extract preparation as described above. For pulldown assays via PTP tag, cell extracts were incubated at 4°C with 25 μl packed IgG Sepharose 6 Fast Flow beads (Invitrogen), equilibrated in IPP-150 buffer (150 mM KCl, 20 mM Tris–Cl, pH 7.7, 3 mM MgCl_2_, 0.5 mM DTT, 0.1% Tween-20). After washing with the same buffer (or with IPP-500, which contains 500 mM KCl), coselected RNAs were released by proteinase K buffer treatment and analyzed by RT-PCR. Amplification products were analyzed by agarose gel electrophoresis.

### Antibodies and immunoprecipitation analysis

The open reading frame of *T. brucei* U1C was PCR amplified from genomic DNA and cloned into pGEX-6P-2. Recombinant proteins were expressed with an N-terminal glutathione *S-*transferase (GST) tag in *Escherichia coli* BL21(DE3)pLys and purified by glutathione affinity chromatography on an ÄKTApurifier high-pressure liquid chromatography system (GE Healthcare). Purified proteins were then used to immunize rabbits (SeqLab, Germany). The resulting immune sera were depleted of GST-reactive antibodies with immobilized GST and affinity-purified, using recombinant expressed GST-U1C. Anti-TbU1-70K antibodies were described previously (5), and immunoprecipitations were performed according to Jaé *et al.* (28).

## RESULTS

### Nuclear localization of *T. brucei* U1C

**Figure 1.**
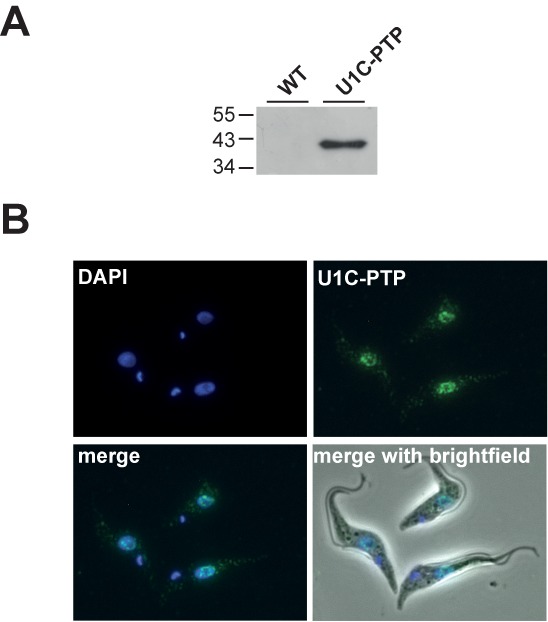
Expression and nuclear localization of *Trypanosoma brucei* U1C-PTP. (**A**) Expression of U1C-PTP was controlled by western blotting with polyclonal antibodies against the protein A epitope of the PTP tag, comparing wild-type (WT) and U1C-PTP expressing cells (U1C-PTP). Protein size markers in kDa. (**B**) *T. brucei* cells stably expressing U1C-PTP were fixed and stained with DAPI (DAPI). In parallel, PTP-tagged U1C was detected by anti-protein A primary antibody and Alexa-Fluor-488-coupled secondary antibody (U1C-PTP). In addition, merged views are shown (‘merge’: DAPI and U1C-PTP staining; ‘merge with brightfield’).

To investigate cellular localization and genome-wide RNA binding of the *T. brucei* U1C protein, we first generated a clonal procyclic cell line, which stably expresses U1C with a C-terminal PTP tag, consisting of two protein A epitopes, a TEV-cleavage site and a protein C epitope (24). U1C expression was monitored by western blot analysis, and the cellular distribution of U1C was characterized by indirect immunofluorescence (Figure 1). U1C-PTP predominantly localizes to the nucleus, with only minor staining of the cytoplasm (Figure 1B), consistent with other spliceosomal components in *T. brucei* (e.g. see (26,29)).

### *In vivo* RNA-crosslink sites of U1C and U1-70K reveal a potential physical link between *cis*- and *trans*-spliceosomal components

**Figure 2.**
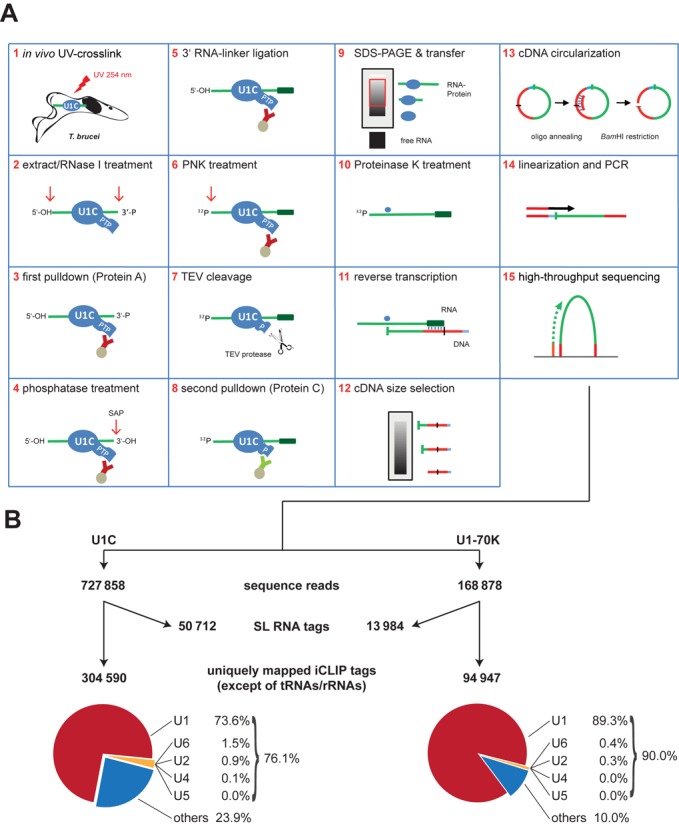
Genome-wide mapping of U1C and U1-70K RNA–protein interactions in trypanosomes by iCLIP-Seq: strategy and statistics. (**A**) Schematic overview of the iCLIP-Seq approach, as adapted for *Trypanosoma brucei* cell lines stably expressing PTP-tagged RNA-binding proteins (here: U1C). For a detailed description, see ‘Results’ section. (**B**) Summary of distribution of U1C and U1-70K iCLIP tags. The numbers of sequence reads, of uniquely mapped reads for the Tb427 genome and of the separately aligned SL RNA tags represent the sum of three (U1C) or two (U1-70K) biological replicates (for the numbers of the individual experiments, see Supplementary Figure S1). The pie charts show the distribution of uniquely mapped reads in snRNAs (U1, U2, U4, U5 and U6) and genomic regions other than snRNA and SL RNA loci (‘others’).

To search for potential new functions of the trypanosome U1 snRNP, we next identified RNA interactions of the U1C protein: we adapted iCLIP in combination with deep-sequencing [iCLIP-Seq; (23)] to the trypanosome system (Figure 2A). For comparison, iCLIP-Seq was performed in parallel with U1-70K, another U1 snRNP-specific protein component. We made use of the highly efficient, two-step affinity purification of RNA–protein complexes, based on a PTP-tagged, stably expressed protein. Briefly, after UV-mediated *in vivo* crosslinking (Figure 2A), cell lysis, limited RNase digestion and the first step of the tandem-affinity purification (steps #1–3), we performed the subsequent steps of the iCLIP procedure on beads, including phosphatase treatment, 3′-RNA linker ligation and polynucleotide kinase treatment (steps #4–6). Next, we used TEV protease to release bound material from the beads (step #7) and applied the second step of the tandem-affinity procedure, anti-protein C immunoaffinity purification (step #8). Purified RNA–protein complexes were subjected to SDS-PAGE, followed by electro-blotting (step #9). Complexes were then eluted by proteinase K treatment (step #10). After reverse transcription (RT) and cDNA size selection (steps #11–12), cDNAs were circularized and BamHI-linearized, followed by PCR addition of sequencing adapters (steps #13–14) and sequencing (step #15).

Five independent iCLIP experiments, three for U1C and two for U1-70K, were performed and sequenced by Illumina GAIIx, Illumina MiSeq and Ion Torrent PGM [see Figure 2B for a summary of the three U1C (left) and the two U1-70K experiments (right); for separate analyses of each individual experiment, see Supplementary Figure S1]. This yielded a total of ∼1.4 million single-end sequence reads. Following barcode removal and 3′ linker trimming, ∼900 000 sequence reads (∼728 000 for U1C, ∼169 000 for U1-70K) with a minimum length of 15 bp were aligned to the Tb427 genome. Uniquely mapped sequence reads, excluding those mapped to tRNAs and rRNAs were selected as iCLIP tags for downstream analysis (for details, see ‘Materials and Methods’ section). Due to the multi-copy array of the SL RNA locus, the sequence reads were aligned separately to the 139-nucleotide SL RNA sequence to determine the iCLIP tags on this RNA.

In summary, ∼305 000 tags for U1C and ∼95 000 tags for U1-70K were selected to identify the RNA-binding sites for the two U1 snRNP proteins on a genome-wide level. In addition, there were ∼51 000 U1C and ∼14 000 U1-70K tags on the SL RNA.

**Figure 3.**
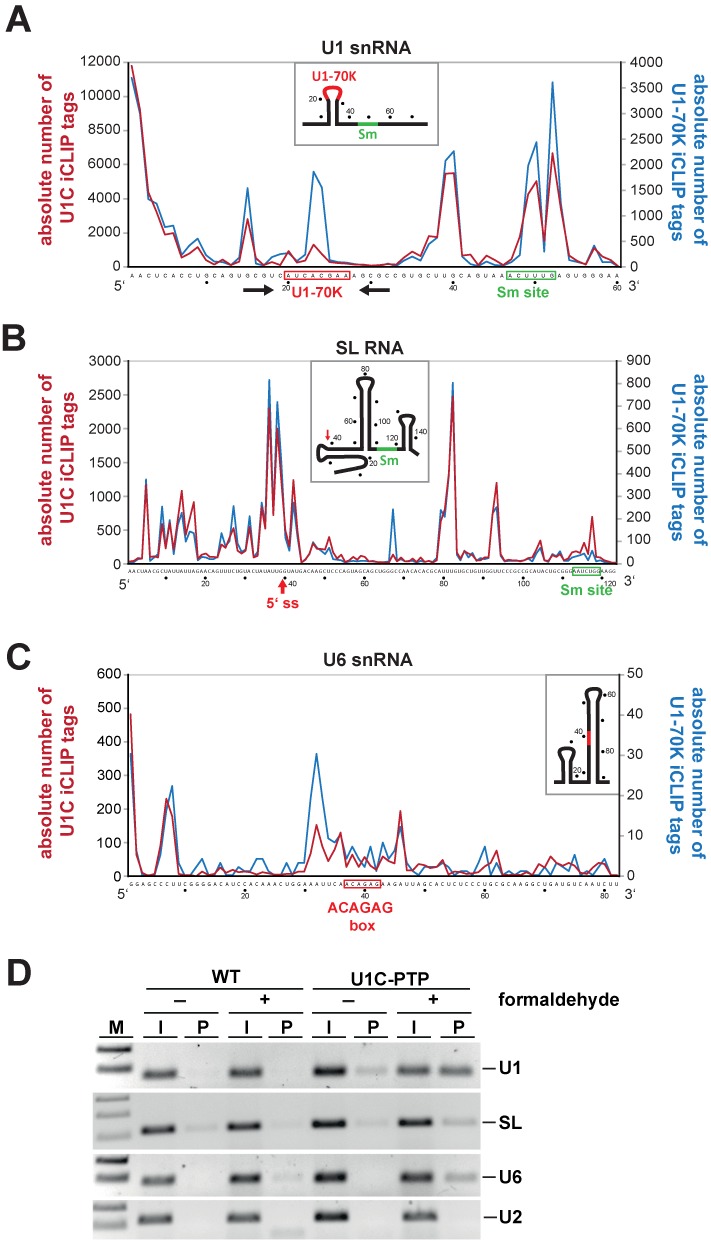
Crosslink site profiles of *Trypanosoma brucei* U1C and U1-70K on the U1, SL and U6 snRNAs. (A–C) The numbers of random-barcode-filtered iCLIP tag counts for U1C (red line) and U1-70K (blue line) iCLIP tags (crosslink sites) on the U1, SL and U6 snRNAs are plotted in single-nucleotide resolution. Only truncated versions of the entire RNA sequences without the last 15 nucleotides are shown (U1 snRNA: nucleotides 1–60; SL RNA: 1–123; U6 snRNA: 1–82), since for technical reasons iCLIP tags further 3′ cannot be mapped. Schematic models of the secondary structures are depicted for each RNA. (**A**) iCLIP profiles on the U1 snRNA. The U1-70K (red) and the Sm binding sites (green) are boxed, the stem-loop structure is indicated by arrows. (**B**) iCLIP profiles on the SL RNA. The 5′ splice site (5′ss; after position 39) is highlighted by a red arrow, the Sm site boxed in green. (**C**) iCLIP profiles on the U6 snRNA. The highly conserved ACAGAG hexanucleotide, which interacts with the 5′ splice site, is boxed in red. (**D**) Validations of U1C iCLIP tags for the U1, SL and U6 snRNAs. Cell extracts were prepared from *T. brucei* wild-type (WT) cells and a cell line stably expressing PTP-tagged U1C, without (−) and with (+) prior crosslinking by formaldehyde. Extracts were subjected to IgG pulldown of PTP-tagged complexes, followed by crosslink reversal. Copurifying U1, SL and U6 snRNAs (as indicated on the right) were detected by RT-PCR (lanes P). For comparison, 1% of the total input is shown (lanes I). M, markers (100 and 200 bp; for panel SL: 100, 200 and 300 bp).

In each of these five experiments, crosslink sites were most abundant in the U1 snRNA, representing 74% (U1C) and 89% (U1-70K) of the total uniquely mapped tags. To examine the crosslink-site profile on a single-nucleotide resolution, random-barcode-filtered tag counts were plotted on the Y-axis for each U1 snRNA nucleotide position (X-axis). Figure 3A shows the U1 snRNA profile derived from the sum of three U1C and two U1-70K iCLIP experiments. Supplementary Figure S2 shows the profiles for the individual replicate experiments, indicating that the iCLIP tag profiles for the U1 snRNA are highly reproducible.

For U1C, ∼40% of the crosslink sites map in the 5′ terminal region (nucleotides 1–9; Figure 3A), consistent with the known binding site of U1C (5). The remaining sites distributed throughout the U1 snRNA sequence, with four peaks at positions 15, 39/40, 50 and 53. Surprisingly, the crosslink profiles for U1C and U1-70K closely resemble each other, except for a U1-70K-specific peak at positions 23/24 (Figure 3A). This is exactly in the central loop of U1 snRNA, which contains the highly conserved U1-70K binding site AUCACGAA (nucleotides 20–27), confirming our earlier *in vitro* binding data (5). To investigate whether the three downstream peaks reflect additional interactions of U1C/U1-70K within the U1 snRNA or crosslink sites of the other U1 snRNA-associated proteins, we performed an additional control: limited RNase digestions indicated that the U1 snRNA stayed largely intact at the RNase concentrations applied during iCLIP, whereas other snRNAs were already partially degraded (data not shown); this suggests that the *T. brucei* U1 snRNP with its unusually short U1 snRNA (75 nucleotides) is highly compact and relatively stable to RNase digestion (see also ‘Discussion’ section).

There is also a surprisingly high number of crosslinks in the SL RNA: about 50 000 for U1C and ∼14 000 for U1-70K (Figure 2B). As seen for the U1 snRNA, the crosslink site profiles of U1C and U1-70K on the SL RNA revealed a very similar distribution (Figure 3B), in particular around the 5′ splice site (triple peak at positions 36–43), suggesting the entire U1 snRNP engages in the recognition or activation of the SL RNA 5′ splice site. There is a second, broad peak around the central loop of the SL RNA (nucleotides 79–82). However, some minor differences were also detected: U1-70K shows an additional peak at position 67, which is absent for U1C, indicating a U1-70K-specific contact with the SL RNA.

We also mapped iCLIP tags on the U6 snRNA, where—following the U1 and SL snRNAs—most crosslink tags had been found (Figures 2B and 3C). In addition to the 5′ terminal nucleotides, the two strongest peaks flank the highly conserved ACAGAG hexanucleotide of U6 snRNA (nucleotides 37–42), which interacts with the 5′ splice site during spliceosome assembly. Significantly, the U1-70K crosslink sites concentrate directly upstream of the ACAGAG box (around position 32). In contrast, only relatively few or no crosslinks could be mapped to the other spliceosomal snRNAs, U2, U4 and U5.

To validate these interactions, we used our procyclic *T. brucei* cell line, which stably expresses PTP-tagged U1C: RNA–protein and protein–protein complexes were formaldehyde-crosslinked *in vivo*, followed by lysate preparation and purification of U1C-containing RNPs, based on the first step of the tandem-affinity purification. After crosslink reversal and RT-PCR assays, signals for U1, SL and U6 snRNAs were detected, but not for U2 snRNA (Figure 3D), confirming specific associations between U1C protein and the U1, SL and U6 snRNAs. In sum, our genome-wide iCLIP data provide direct evidence for physical links between *cis*-splicing components (U1C; U1 snRNP) and the *trans*-splicing machinery (SL RNA).

### The 5′ splice site of *cis*-spliced *PAP* pre-mRNA is recognized by U1C and U1-70K

**Figure 4.**
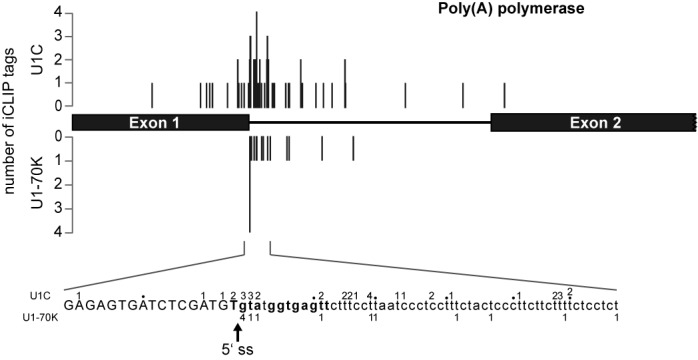
Crosslink site profiles of *Trypanosoma brucei* U1C and U1-70K on the poly(A) polymerase (*PAP*) pre-mRNA. The numbers of random-barcode-filtered iCLIP tags for U1C and U1-70K are plotted on the Y-axis with the corresponding crosslink sites. Shown are the exon–intron–exon region of the *PAP* pre-mRNA (top) and detailed information on the absolute number of CLIP tags at nucleotide resolution for the 5′ splice site region (bottom; U1C above, U1-70K below the sequence); the arrow marks the 5′ splice site (5′ ss).

Because U1C is thought to be involved in 5′ splice site recognition, we next analyzed crosslink sites on the two *cis*-spliced pre-mRNAs, *PAP* (Tb927.3.3160) and an ATP-dependent DEAD Box helicase (Tb927.8.1510). The well-characterized *PAP* gene was described as the first protein-coding gene in *T. brucei* that contains an intron and requires processing of its primary transcript through *cis*-splicing (30). Clearly, both U1C and U1-70K binding sites cluster around the *PAP* 5′ splice site (positions −1 to +3) (Figure 4). A second, U1C-specific cluster maps to a more downstream region (+15 to +19).

For the second intron-containing gene, coding for an ATP-dependent DEAD Box helicase, only few U1C crosslink sites were identified, probably due to its lower expression levels. However, 5′ splice site interaction can be clearly seen for both U1 snRNP proteins (positions +11 and +22 relative to the 5′ splice site) (Supplementary Figure S6A).

In sum, we conclude that the 5′ splice site of both *cis*-spliced pre-mRNAs is contacted *in vivo* by both U1C and U1-70K proteins, and therefore most likely recognized by the entire U1 snRNP.

### U1C depletion decreases the efficiency of *cis*-splicing and becomes essential under stress conditions

**Figure 5.**
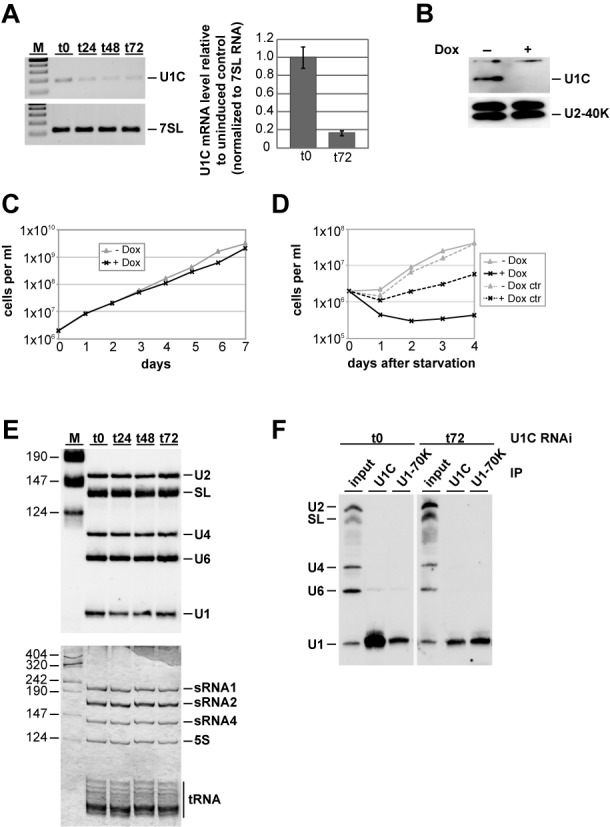
Effects of RNAi-mediated depletion of *Trypanosoma brucei* U1C on growth and U1 snRNP integrity. (A, B) RNAi-mediated knockdown of U1C expression. 24, 48 or 72 h after RNAi induction (as indicated), RNA was analyzed by semiquantitative RT-PCR (top) and real-time PCR (bottom). In addition, RNA from uninduced cells (t0) was included, and, as a control, 7SL RNA was measured from the same RNA samples (**A**). M, markers (100, 200, 300, 400 and 500 bp). In parallel, U1C protein was detected by immunoblotting with affinity-purified polyclonal antibodies, using U2-40K protein as a control (**B**). (**C**) Growth curve of a representative clonal procyclic *T. brucei* cell line, in which RNAi against the U1C mRNA was induced. Cells were grown for 7 days in the absence (−Dox; gray line with triangles) or in the presence of doxycyline (+Dox; black line with crosses), counted every day, and diluted back to 2 × 10^6^ cells/ml. (**D**) Recovery of procyclic cells with or without induction of U1C RNAi from starvation stress. Cells were grown for 1 day in the absence (−Dox) or in the presence of 1 mg/ml doxycycline (+Dox). As a control for growth effects of doxycycline, control cells (ctr) were grown for 1 day under the same conditions. 2 × 10^6^ cells/ml were collected, washed twice in phosphate-buffered saline (PBS), resuspended in the original volume of PBS, and incubated at 27°C for 2 h. After starvation stress, cells were harvested again, resuspended in the original volume SDM-79, and grown in the absence (−Dox; gray line with triangles) or in the presence of doxycyline (+Dox; black line with crosses). (**E**) U1C knockdown does not affect snRNA steady-state levels. From uninduced cells (t0) and 24, 48 and 72 h after silencing of U1C expression, equal amounts of RNA were analyzed by northern blot hybridization, using a mixed probe for U2, SL, U4, U6 and U1 snRNAs (top panel). As a loading control, equal amounts of RNAs were detected by silver staining (bottom panel). Positions of snRNAs, ribosomal RNAs and tRNAs are marked on the right. M, markers (in nucleotides). (**F**) Immunoprecipitation analysis of U1 snRNPs upon U1C knockdown. From uninduced cells (t0) and 72 h after U1C knockdown (t72), whole-cell extracts were prepared and subjected to immunoprecipitation (IP), using anti-U1C or U1-70K antibodies (as indicated). Copurifying RNAs were analyzed by northern blotting, using a mixed probe for U2, SL, U4, U6 and U1 snRNAs (positions indicated on the left). For comparison, 5% of each input is shown (lanes ‘input’).

To further evaluate the functional role of U1C during splicing, we silenced U1C expression by RNAi (Figure 5). Efficient knockdown was confirmed by RT-PCR detection of U1C mRNA, using 7SL as loading control; already after day 1, U1C mRNA levels decreased to half. We also performed qPCR using the same primer pairs and observed a knockdown efficiency of approximately 80% after 3 days (Figure 5A). In addition, we checked for efficient depletion of U1C protein by western blot: after 3 days of knockdown, U1C protein was almost undetectable (Figure 5B). To address the question whether U1C is required for cell viability, we depleted cells of U1C during a time period of 7 days. Surprisingly, no significant difference in growth between induced and uninduced cells could be observed, suggesting that U1C may not be essential for cell viability of procyclic *T. brucei* under the conditions used here (Figure 5C). To examine whether this might be different under certain stress conditions, we exposed U1C-depleted trypanosome cells to starvation stress and measured by their growth how they recovered (Figure 5D): cells, in which RNAi-knockdown of U1C had been induced, failed to recover from the starvation stress, whereas cells without nutrient starvation started to recover after 1 day.

Next, we asked whether the steady-state levels of the snRNAs are affected by the RNAi-mediated knockdown. U1C expression was silenced by RNAi for 72 h, and the steady-state levels of the SL, U1, U2, U4 and U6 snRNAs were analyzed by northern blot hybridization during this time period; as an input control, ribosomal RNAs were detected by silver staining (Figure 5E). Neither the U1 snRNA nor any of the other snRNAs were affected by U1C knockdown (Figure 5E). In addition, we checked for the integrity of the U1 snRNP, using anti-U1C or anti-U1-70K immunoprecipitation (Figure 5F). In uninduced cells, the efficiencies of U1 snRNA immunoprecipitation by anti-U1C and anti-U1-70K antibodies were ∼50 and ∼10%, respectively. Upon U1C knockdown for 72 h, anti-U1C immunoprecipitation efficiency decreased to approximately 5–10%, whereas the corresponding value for U1-70K remained unchanged. We conclude that U1C protein depletion did not affect snRNA steady-state levels nor U1 snRNP integrity.

**Figure 6.**
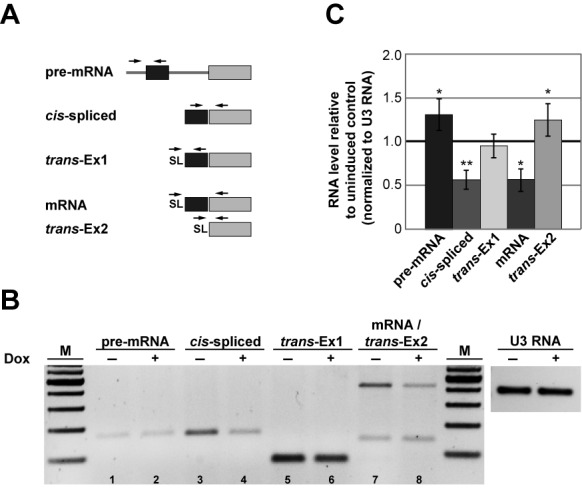
U1C depletion decreases the efficiency of *cis*-splicing. (**A**) Schematic overview of the primer combinations used to detect *PAP* [poly(A) polymerase, Tb927.3.3160] pre-mRNA and mRNA by combinations of exon-, intergenic-region, intron- or SL-specific primers. The same primer pairs detected both *cis*-spliced and *cis*-unspliced products, as well as *trans*-spliced mRNA (mRNA) and aberrant *trans-*splicing at exon 2 (*trans*-Ex2). (**B**) Inhibition of *cis*-splicing by U1C knockdown. Total RNA from uninduced (−) and induced (+) cells after 72 h were analyzed by semiquantitative RT-PCR, using the primer combinations described in panel (A). As a control, U3 RNA was measured from the same RNA samples. M, markers (100, 200, 300, 400, 500, 600 and 700 bp). (**C**) Quantification of RT-PCR reactions shown in panel (B). Error bars represent standard deviations from three independent experiments. *P < 0.05 and **P < 0.01 versus uninduced control.

To investigate whether U1C is essential for splicing *in vivo*, we analyzed *cis* splicing by semiquantitative RT-PCR, using various primer combinations to detect pre-mRNA and splicing products, and normalizing to U3 RNA expression (Figure 6). Upon U1C knockdown we observed *cis*-splicing defects for the *PAP* and the ATP-dependent DEAD Box helicase pre-mRNAs (Supplementary Figure S6B–D**)**: Specifically, *PAP* pre-mRNA accumulated (Figure 6B; lanes 1/2), whereas *cis*-spliced product clearly decreased upon U1C knockdown, indicating a block of *cis-*splicing (lanes 3/4), consistent with the effect on mature mRNA detected by an SL–exon 2 primer combination (mRNA; lanes 7/8, upper band). In contrast, no change in *trans*-splicing efficiency was detected for the same gene (*trans*-Ex1; lanes 5/6). Interestingly, if *cis*-splicing was inhibited by RNAi depletion of U1C, the exon 2 of the *PAP* pre-mRNA was subjected to *trans-*splicing more frequently than in uninduced cells (*trans*-Ex2; lanes 7/8, lower band).

As an alternative way of inhibiting the activity of the U1 snRNP, we also used an antisense morpholino oligonucleotide (AMO) that specifically blocks the 5′ end of the U1 snRNA, as shown by its ability to specifically select U1 snRNA in total RNA (Supplementary Figure S3A). Following transfection of *T. brucei* cells with the U1-specific versus a control AMO, we analyzed after a 6-h incubation splicing of the *PAP* gene by RT-PCR, using different primer combinations (Supplementary Figures S3B–D). Upon AMO inhibition of the U1 snRNP, we observed splicing defects comparable to those seen after U1C depletion by RNAi: in addition to pre-mRNA accumulation, an increase of unspliced product was detected (5′ unspliced and *cis* unspliced). In contrast to the RNAi effect, mature *cis*-spliced mRNA signals did not change significantly, probably due to the relatively short period of AMO incubation.

Taken together, it appears that surprisingly, RNAi-mediated depletion of U1C does not significantly affect parasite growth under normal conditions, nor does it alter the U1 snRNP integrity; we observe only moderate effects on *cis*-splicing, but no effect on normal SL *trans*-splicing. In contrast, recovery from starvation stress was clearly impeded by U1C depletion, indicating that *cis*-splicing becomes essential only under stress conditions.

## DISCUSSION

To analyze RNA–protein interaction in the trypanosome system on a genome-wide level, we adapted the iCLIP approach (23). The major change concerned the initial immunoprecipitation stage, where we made use of the highly efficient tandem-affinity purification technology, combined with *T. brucei* cell lines that stably express PTP-tagged proteins of interest. In our experience such an initial two-step, high-affinity purification greatly helps to generate CLIP libraries with very low background, compared with standard, single-step immunoprecipitations. However, one should also consider that the two-step affinity purification can result in relatively low yields and therefore correspondingly low quantities of final iCLIP tags, in particular for low-abundance proteins. Here, we analyzed U1C and U1-70K, two specific protein components of the U1 snRNP. Initially, we focused on the U1C protein, which, based on studies in other systems, is particularly important for the correct recognition of the 5′ splice site (see ‘Introduction’ section for references). U1-70K, another U1-specific component with a highly conserved RNA-binding site in the first loop of U1 snRNA, primarily served as an internal specificity control and for direct comparison.

After evaluating iCLIP patterns for U1C and U1-70K, including additional control experiments, we conclude that the iCLIP approach reaches a certain limit, when analyzing relatively small RNPs with multiple protein components and protein–protein interactions, such as the trypanosomal U1 snRNP, with its 75-nts snRNA component and a total of 11 proteins. In such cases, fragmentation by RNase during the iCLIP procedure may not be sufficient and may result in RNA fragments with more than a single polypeptide covalently crosslinked, up to the complete small RNP with several crosslinks. As a result of such partial or complete RNase resistance, we observe multiple peaks in the iCLIP profile. This reflects RNA–protein interactions throughout the U1 snRNP, due to coprecipitation effects and ‘RNA linkers’ between polypeptides that are too short for effective fragmentation. For example, we see an additional double peak (nucleotides 50/53 of the U1 snRNA) around the Sm site (5′-ACUUUG-3′), most likely due to RNA contacts with one of the Sm polypeptides, which interacts with U1C, as shown for SmD3 in the human U1 snRNP (16). On the other hand, there are still protein-specific crosslink peaks, which are due to partial fragmentation of the RNP in this region, e.g. for U1-70K (at nucleotides 23/24 in the central loop). Finally, we cannot assign two other prominent peaks upstream (at nucleotides 15 and 39/40), which suggest additional protein contacts, e.g. by the U1 snRNP components U1A or U1-24K.

In spite of these intrinsic limitations, which should apply also to CLIP analyses of other small RNPs, RNA–protein interaction maps can be deduced, which allow insights into spliceosomal networks on the level of individual snRNAs and specific proteins. Due to spliceosomal dynamics, we have to be aware that we observe the sum of various conformational states with different occupancies. Focusing here on U1C and U1-70K, we found—in addition to U1 snRNA—iCLIP tags also in the SL RNA and the U6 snRNA.

We were very surprised that most of the U1C/U1-70K crosslinks on the SL RNA map to its 5′ splice site, because this suggests that the U1 snRNP participates in the recognition and/or activation of the 5′ splice site common to all *trans*-splicing reactions. U1C may be directly involved in the SL 5′ splice site activation, and—because we see the same crosslink pattern for U1-70K—it is likely the entire U1 snRNP that interacts. Because—except for the Sm proteins—no other SL RNP proteins are known in trypanosomes, the other major RNA–protein contact on the SL RNA (nucleotides 79–83), detected for both U1C and U1-70K, most likely indicates a novel protein bound at the central loop and interacting with U1C/U1-70K.

In the case of the two known *cis*-spliced 5′ splice sites (*PAP* and DEAD Box RNA helicase), the iCLIP crosslink patterns clearly peak in the first intronic positions of the 5′ splice sites. In particular, this was shown for both U1C and U1-70K sequence tags, which peak in the first intronic positions of the *PAP* 5′ splice site, suggesting a direct involvement of the U1 snRNP in 5′ splice site recognition.

Finally, when comparing the iCLIP patterns for the snRNAs and the *PAP* 5′ splice site, we note that the absolute number of crosslink tags differs within a range of three orders of magnitude (compare Figures 3 and 4). This can be explained not only by different expression levels of the respective target RNAs, but also by the transient versus stable nature of certain RNA–protein interaction and corresponding occupancy times.

**Figure 7.**
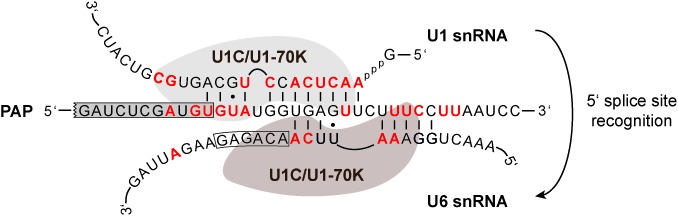
Model of 5′ splice site recognition in the *PAP* pre-mRNA and summary of U1C/U1-70K iCLIP tags. This schematic shows how the 5′ splice site in the *cis*-intron of the poly(A) polymerase (*PAP*) pre-mRNA can be sequentially recognized by the U1 snRNA (5′ terminal region) and the U6 snRNA (internal region), based on the proposed U1 snRNA (27) and a hypothetical, extended U6 snRNA base-pairing (this study). Boxed are the exonic nucleotides of the *PAP* pre-mRNA and the conserved ACAGAG sequence of the U6 snRNA. The iCLIP-derived U1C/U1-70K-crosslinked nucleotides in all three RNAs (U1, U6 and *PAP*) are highlighted in red, to allow direct comparison with the base-pairing interactions.

We summarize these crosslink characteristics for the *PAP cis*-intron, the U1 and U6 snRNAs in a model, focusing on U1C/U1-70K and taking into account our knowledge of spliceosomal dynamics established in the yeast and mammalian systems (see Figure 7). Early in spliceosome assembly, the U1 snRNA extensively base-pairs with the *PAP* 5′ splice site, as proposed by Mair *et al.* (30) and supported here by directly adjacent crosslink positions in the *PAP* intron and the U1 snRNA (see nucleotides marked in red). Later, U6 snRNA replaces U1, and we propose this extended base-pairing, based on the classical register between the U6 ACAGAG sequence and the first intronic positions of the 5′ splice site. The crosslink patterns derived from our iCLIP data and summarized in Figure 7 support that such a sequential, multiple 5′ splice site recognition and U1-to-U6 ‘handing-over’ operates also in the trypanosome system.

A surprising implication of our first iCLIP study in the trypanosome system is that it suggests a physical linkage between *cis*- and *trans*-splicing, in particular the U1 snRNP and the SL RNP: the 5′ splice sites of both the *PAP cis*-intron and the *trans*-spliceosomal SL RNA are contacted by U1C and U1-70K. Based on this, the most provocative hypothesis would be that the U1 snRNP participates in activation of the SL RNA 5′ splice site. This would be in contrast to results in the nematode system, where U1 snRNA appears not to be essential for *trans-*splicing: first, knockout of over 90% of U1 snRNA did not affect *trans-*splicing *in vitro* (31); second, only traces of U1 snRNA were detectable in purified *trans* spliceosomes, probably due to cryptic 5′ splice sites (32). Third, as proposed by Bruzik *et al.* (33), the SL RNA may use its base-paired structure around the 5′ splice site to be activated in a U1-independent manner. However, note that we cannot be certain that the nematode results apply to the trypanosome system, with its highly divergent U1 snRNA and very few *cis* introns.

On the other hand, the SL RNA is clearly present in both nematode *cis*- and *trans*-spliceosomes (32), suggesting a common *cis*/*trans*-spliceosome, a close linkage between the two types of splicing reactions, and that the decision between *cis*- and *trans*-splicing occurs after spliceosome assembly. We consider such a scenario likely to operate also in trypanosomes, and our result on the competitive usage of the 3′ splice site in the *PAP* intron supports this notion: upon U1C knockdown we detected a partial switch of the *PAP* 3′ splice site from normal *cis*- to aberrant *trans*-splicing (Figure 6B, lanes 7/8).

Why did we not observe a severe growth defect after U1C (Figure 5) nor U1-70K knockdown (Supplementary Figure S4) under ‘normal’ conditions? First, the U1 snRNP and the U1C protein are apparently not essential for *trans-*splicing in trypanosomes, at least under normal conditions, consistent with earlier nematode studies (see above). The U1C/U1-70K contact with the SL 5′ splice site we described may be functionally relevant only under certain growth conditions, such as during stress, or for a subset of genes. However, a high-throughput phenotype analysis revealed that for either of the U1 snRNP-specific proteins U1C and U1-70K no significant loss of fitness was observed upon knockdown in the different life cycle stages (34). Alternatively, it may simply represent a minor specificity or efficiency determinant not significantly relevant for normal growth nor splicing activity under our experimental conditions.

Second, regarding the two *cis*-introns of *T. brucei*, their splicing efficiency was clearly affected upon U1C knockdown, although only moderately, consistent with an earlier report on U1-70K knockdown (19). The intrinsic inefficiency of *cis*-splicing in trypanosomes can explain the moderate extent of this effect: already under normal growth conditions a high number of intronic reads was measured by RNA-Seq, reflecting unspliced, intron-containing pre-mRNA (Supplementary Figure S5). Why did we not detect any significant defect in growth after knockdown? We suggest that there is redundancy among several paralogous poly(A) polymerases in the genome, or that even the intron-containing *PAP* (Tb927.3.3160) may not represent the major functional gene responsible for mRNA 3′ end processing. The same argument holds for the second intron-containing gene (Tb927.8.1510), coding for a putative RNA helicase of unknown functionality and specificity. This hypothesis is further supported by the high-throughput RNAi analysis of Alsford *et al.*, in which knockdown of neither of these two genes showed a severe effect (34).

However, when exposing U1C-depleted cells to starvation stress, a severe growth defect was observed, indicating that U1C and *cis*-splicing become essential in the response to nutrient starvation.

Ultimately, this points to the possibility that *cis*-splicing of the two introns itself may not be required for growth of the parasite under ‘standard’ conditions. For both intron-containing genes functional intronless putative counterparts may exist (for the poly(A) polymerase: Tb927.3.3160/Tb927.7.3780; for the ATP-dependent DEAD Box helicase ATP: Tb927.8.1510/Tb927.10.6630), so that the spliceosomal U1 snRNP may represent an evolutionary relic. However, this does not exclude that the two introns may fulfill an unknown important function, and that other U1 RNPs with variant protein compositions play roles beyond splicing.

## Accession Number

NCBI GEO database: GSE43848.

## SUPPLEMENTARY DATA

Supplementary Data are available at NAR Online.

SUPPLEMENTARY DATA
