# Supplementary material for: Genome-wide RNA-binding analysis of the trypanosome U1 snRNP proteins U1C and U1-70K reveals cis/trans-spliceosomal network
Source: Nucleic Acids Res. 2014 Apr 19;42(10):6603–15. doi: 10.1093/nar/gku286 (PMC4041458; doi:10.1093/nar/gku286)
Supplement: SUPPLEMENTARY DATA [file supp_42_10_6603__index.html]

Genome-wide RNA-binding analysis of the trypanosome U1 snRNP proteins U1C and U1-70K reveals cis/trans-spliceosomal network — Genome-wide RNA-binding analysis of the trypanosome U1 snRNP proteins U1C and U1-70K reveals cis/trans-spliceosomal network — SUPPLEMENTARY DATA 

# Genome-wide RNA-binding analysis of the trypanosome U1 snRNP proteins U1C and U1-70K reveals *cis*/*trans*-spliceosomal network

## SUPPLEMENTARY DATA

**Files in this Data Supplement:**

- SUPPLEMENTARY DATA
