# Supplementary material for: Genome-wide RNA-binding analysis of the trypanosome U1 snRNP proteins U1C and U1-70K reveals cis/trans-spliceosomal network
Source: Nucleic Acids Res. 2014 Apr 19;42(10):6603–15. doi: 10.1093/nar/gku286 (PMC4041458; doi:10.1093/nar/gku286)

*SUPPLEMENT*

# Genome-wide RNA-binding analysis of the trypanosome U1 snRNP proteins U1C and U1-70K reveals *cis*/*trans*-spliceosomal network

Christian Preußer, Oliver Rossbach, Lee-Hsueh Hung, Dan Li, and
Albrecht Bindereif,*

Institute of Biochemistry, Justus Liebig University of Giessen, D-35392 Giessen, Germany

* To whom correspondence should be addressed. Tel: 49-641-9935 420; Fax: 49-641-9935 419; Email: albrecht.bindereif@chemie.bio.uni-giessen.de

The authors wish it to be known that, in their opinion, the first three authors should be regarded as joint First Authors

## SUPPLEMENTARY MATERIALS AND METHODS

### Antisense morpholino transfection

For antisense morpholino transfection with either 10 µM or 100 µM antisense morpholino oligonucleotide (U1 5′-GGTATCTCCCCTGCCAGGTAAGTAT-3′ and Ctr 5′-GGTATCTCCCCTGCCAGGTAAGTAT-3), *T.brucei* 29-13 cells in the mid-log phase (2x$10^6$ cells per ml) were transfected by electroporation. The efficiency of the transfection was assessed by immunofluorescence. The specificity of the morpholino oligonucleotide was assessed by streptavidin selection. Cells were washed in 1x PBS, followed by extract preparation as described in the main text. For streptavidin selection, cell extracts were incubated at 4°C with pre-blocked 25 µl packed Neutravidin beads (Invitrogen), equilibrated in WB500 buffer (500 mM KCl, 20 mM Tris-Cl, pH 7.7, 1 mM DTT, 0.01% NP40). After washing with the same buffer (or with WB50, which contains 50 mM KCl), coselected RNAs were released by proteinase K buffer treatment and analyzed by RT-PCR, using primers for U1 and U2 snRNAs. Amplification products were analyzed by agarose gel electrophoresis.

Semiquantitative RT-PCR was performed as described (26), and amplification products were characterized by an Agilent Bioanalyzer 2100, using DNA 1000 Lab-Chip kits.

### RNA-Seq sample preparation

For non-strand sensitive high-throughput sequencing, poly(A)-selected RNA was processed by TruSeq RNA Sample Preparation Kit (Illumina) to prepare the mRNA-Seq library, followed by sequencing in a paired-end, 101-base mode on a HiSeq 2000. RNA-Seq sequence-read data were uploaded to the Sequence Read Archive at NCBI (SRX444324).

### Oligonucleotides

DNA oligonucleotides (MWG Eurofins) are listed in the following.

For preparing DIG-labeled snRNA-specific probes:

| | |
|---|---|
| SP6-TbU1-Fw: | 5'-ATTTAGGTGACACTATAGAACTCACCTGCAGTGCGT-3' |
| TbU1-Rev: | 5'-AGGGACGCTTTCGTTCCC-3' |
| TbU2-Fw: | 5'-ATATCTTCTCGGCTATTTAGC-3' |
| TbU2-Rev: | 5'-ACCGTCGCGCTCCATCC-3' |
| TbU4-Fw: | 5'-AAGCCTTGCGCAGGGAGG-3' |
| TbU4-Rev: | 5'-TACCGGATATAGTATTGCAC-3' |
| TbU6-Fw: | 5'-GGAGCCCTTCGGGGACA-3' |
| TbU6-Rev: | 5'-AAAAGCTATATCTCTCGAAGAT-3' |
| SP6-TbU5-Fw: | 5'-ATTTAGGTGACACTATAGGCATCGCCGTCTCGACTTTTA-3' |
| TbU5-WT-Rev: | 5'-GACACCCCAAAGTTTAAACG-3' |
| SP6-TbSL-Fw: | 5'-ATTTAGGTGACACTATAGAACTAACGCTATTATTAGAACAG-3' |
| TbSL-Rev: | 5'-AAAGAGTGGAGGTCATCCG-3' |

For cloning into pC-PTP-NEO vector:

| | |
|---|---|
| PTP-U1C *Apa*I: | 5'-ATGGGCCCTTTGCTTTGGATGATGCTAGGCGAAT-3' |
| PTP-U1C *Not*I: | 5'-GATCAGCGGCCGCGCTTCACGTTTCGTGACCGAAAGTGG-3' |

For cloning into pGEX-6P-2:

| | |
|---|---|
| GST-U1C fw: | 5'-GCATATGGATCCAGTGATAGTTTTGCTTTGGATGATGC-3' |
| GST-U1C rev: | 5'-AGCGAATTCTCATTCACGTTTCGTGACCGAA-3' |

For generating U1C stem-loop construct:

| | |
|---|---|
| U1C RNAi fw | 5’-GCATAAAGCTTACGCGTCGTGCCCATGTTGAAAATC-3’ |
| U1C RNAi rev | 5’-GCATATCTAGAGAAACTCAATTCTACGGCAAGC-3’ |

For RT-PCR assays:

| | |
|---|---|
| U1C mRNA fw: | 5’-ACGGCACATGGAGAATATGG-3’ |
| U1C mRNA rev: | 5’-TCAACACGTCCAGCAACAAC-3’ |
| SL 6-28: | 5’-ACGCTATTATTAGAACAGTTTCT-3’ |
| PAP Igr fw: | 5’-CCTCCTCCACTTTCCTACGC-3’ |
| PAP Iorf rev1: | 5’-GTTTCGTTGGGCCATACATC-3’ |
| PAP Iorf fw: | 5’-CCTACCCATTTGGTTCATGC-3’ |
| PAP Int rev: | 5’-GAAGAGGACGGGAGAAGAGC-3’ |
| PAP Iorf rev2: | 5’-GGAACTCTGGCAGCGACTAC-3’ |
| PAP Ex2 fw: | 5’-CGATGAGTTTCCGAGGTTGT-3’ |
| Hel Iorf Fw1: | 5’-GCG GGC TTG ACA TTA AGA AC-3’ |
| Hel Int Rv1: | 5’-CGT TGT GGA ATG TGC CTA TG-3’ |
| Hel Int Fw1: | 5’-CCG TTG CTC TCA TTG TGA TG-3’ |
| Hel Iorf Rv2: | 5’-TGG TGG AAT CTC CTG ATT GG-3’ |
| 7SL RNA fw: | 5’-TGACTTGGTGTTCTGCTTGG-3’ |
| 7SL RNA rev: | 5’-CTCGGTGTGCTTCTGCAAC-3’ |
| U3 fw: | 5'-CCGTACTCTGAACAGAATCGTTTT-3' |
| U3 rev: | 5'-CCGTTCATCGAACAGCTCTC-3' |
| SL fw: | 5'-GACACAGCCATGCTTTCAAC-3' |
| SL rev: | 5'-CAGGAACCAACAGCACAATG-3' |
| U1 fw | 5'-ACTCACCTGCAGTGCGTCAT-3' |
| U1 rev | 5'-GACGCTTCCGTTCCCACT-3' |
| U2 fw | 5'-TCGGCTATTTAGCTAAGATCAAGTT-3' |
| U2 rev | 5'-AGTGGAACCCCGGAAGAAC-3' |
| U6 fw | 5'-GGGACATCCACAAACTGGAA-3' |
| U6 rev | 5'-TCTCTCGAAGATTGACATCAGC-3' |

AMO (Gene Tools, LLC):

| | |
|---|---|
| AMO U1 snRNA Tb | 5’-TCGTGATGACGCACTGCAGGTGAGT[Biotin]-3’ |
| AMO ctr | 5’-GGTATCTCCCCTGCCAGGTAAGTAT[Biotin]-3’ |

CLIP RNA 3’-linker (Dharmacon):
5’-P-UGAGAUCGGAAGAGCGGUUCAG-3’ puromycin

iCLIP RT-primers (Eurogentech):

| | |
|---|---|
| iCLIP-RT1: | 5’-NNAACCNNNAGATCGGAAGAGCGTCGTGgatcCTGAACCGC-3’ |
| iCLIP-RT2: | 5’-NNACAANNNAGATCGGAAGAGCGTCGTGgatcCTGAACCGC-3’ |
| iCLIP-RT3: | 5’-NNCTAANNNAGATCGGAAGAGCGTCGTGgatcCTGAACCGC-3’ |
| iCLIP-RT4: | 5’-NNCATTNNNAGATCGGAAGAGCGTCGTGgatcCTGAACCGC-3’ |
| iCLIP-RT5: | 5’-NNGCCANNNAGATCGGAAGAGCGTCGTGgatcCTGAACCGC-3’ |

iCLIP *BamH*I-linearization oligonucleotide (MWG Eurofins):

| | |
|---|---|
| Cut_oligo: | 5’-GTTCAGGATCCACGACGCTCTTCAAAA-3’ |

## SUPPLEMENTARY FIGURE LEGENDS

**Supplementary Figure S1.**
**Summary of distribution of U1C and U1-70K iCLIP tags: individual replicate experiments.**
**(A, B)** The numbers of sequence reads, of uniquely mapped reads, and of the separately aligned SL RNA tags are listed for each individual replicate experiment: U1C (panel A: U1C_1, _2, _3) and U1-70K (panel B: U1-70K_1 and _2). Below, the pie charts represent the distribution of uniquely mapped reads in snRNAs and other regions (in %), separately for individual iCLIP experiments.

**Supplementary Figure S2.**
**Crosslink site profiles of *T. brucei* U1C and U1-70K on the U1 snRNA: individual replicate experiments.**
The numbers of U1C (red line) and U1-70K (blue line) iCLIP tags on the U1 snRNA (nucleotides 1-60) are plotted in single-nucleotide resolution, separately for the individual iCLIP experiments (U1C_1, _2, _3; U1-70K_1 and _2). The U1-70K (red) and the Sm binding sites (green) are boxed, the stem-loop structure is indicated by arrows. Below, a schematic model of the U1 snRNA secondary structure is depicted.

**Supplementary Figure S3.**
**Morpholino-mediated blocking of U1 snRNA inhibits *cis* splicing.**
**(A)** *T. brucei* WT cells were transfected with 10 or 100 µM of a 3'-biotin labeled U1 snRNA antisense morpholino oligonucleotide (AMO; U1) blocking the 5′ end of the U1 snRNA, or with of a control oligonucleotide (ctr; each at 100 µM). Six hours after transfection, whole-cell extract was prepared, and the specificity of the morpholino oligonucleotide was assessed by streptavidin selection. Copurifying RNAs were detected by RT-PCR, using primers for U1 and U2 snRNAs. *M*, markers (bp).
**(B)** Schematic overview of the primer combinations used to detect *PAP* [poly(A) polymerase, Tb927.3.3160] pre-mRNA and mRNA by combinations of exon-, intergenic-region, or intron-specific primers. The same primer pairs detect both *cis*-spliced and *cis*-unspliced products.
**(C)** Inhibition of *cis* splicing upon morpholino-mediated blocking of the U1 snRNA. Total RNA was prepared from cells 6 hours after transfection as described in panel A. Splicing of the *PAP* gene [*poly(A) polymerase,* Tb927.3.3160] was analyzed by RT-PCR. *PAP* pre-mRNA and mRNA were detected by gene-specific combinations of exon, intergenic-region, and intron primers (see panel B; the same primer pairs detect both *cis*-spliced and *cis*-unspliced products). PCR amplification products were characterized by an Agilent 2100 Bioanalyzer. *M*, markers (bp).
**(D)** Quantification of RT-PCR reactions shown in panel C.

**Supplementary Figure S4.**
**Depletion of *T. brucei* U1-70K shows no effect on parasite viability.**
**(A)** RNAi-mediated knockdown of U1-70K expression. U1-70K mRNA was measured by semiquantitative RT-PCR (top) and real-time PCR (bottom) from uninduced cells (t0) and after 72 hours of RNAi induction (as indicated). As a control, U3 (for semi-quantitative RT-PCR) and 7SL RNA (for real-time PCR) were measured from the same RNA samples.
**(B)** Growth curve of a representative clonal procyclic *T. brucei* cell line, in which RNAi-mediated knockdown of U1-70K was induced. Cells were grown for 7 days in the absence (- Dox; grey line with triangles) or in the presence of 1 µg/ml doxycyline

(+ Dox; black line with crosses), counted every day, and diluted back to 2 x $10^6$ cells/ml.

**Supplementary Figure S5.**
**Inefficient *cis*-splicing of the *PAP* gene: RNA-Seq data analysis and read coverage.**
Two independent samples of total RNA extracted from *T. brucei were* poly(A)$^+$-selected. Non-strand-sensitive high-throughput sequencing was performed, using the TruSeq RNA Sample Preparation Kit (Illumina) and a HiSeq 2000 sequencer.. 85 million paired-end sequence reads of 105-bp length from each sample were aligned to the Tb427 genome. Shown here is the read coverage of uniquely mapped sequence reads in the *PAP* gene (Tb427.03.3160; exon1-intron-exon2). High coverage in the intron region indicates inefficient *cis*-splicing.

**Supplementary Figure S6.**
**iCLIP-derived crosslink site profiles of *T. brucei* U1C on the ATP-dependent DEAD box helicase pre-mRNA and splicing inhibition upon U1C depletion.**
**(A)** The distribution of iCLIP crosslink sites of U1C is shown for the exon-intron-exon region of the ATP-dependent DEAD box helicase pre-mRNA (top) as well as detailed information on iCLIP tags at nucleotide resolution for the 5' splice site region; the arrow marks the 5' splice site (5' ss).
**(B)** Schematic overview of the primer combinations used to detect ATP-dependent DEAD box helicase pre-mRNA and mRNA by combinations of exon- or intron-specific primers.
**(C)** Inhibition of *cis*-splicing by U1C knockdown. Total RNA from uninduced (-) and induced (+) cells after 72 hours were analyzed by semiquantitative RT-PCR, using the primer combinations described in panel A. As a control, U3 RNA was measured from the same RNA samples. *M*, markers (100, 200, 300, and 400 bp).
**(D)** Quantification of RT-PCR reactions shown in panel C.

# Supplementary Figure S1

**A**

| | sequence reads | SL RNA tags | uniquely mapped iCLIP tags (except tRNAs/rRNAs) |
|---|---|---|---|
| **U1C_1** | 193,440 | 14,037 | 92,339 |
| **U1C_2** | 290,996 | 27,438 | 151,192 |
| **U1C_3** | 243,422 | 9,237 | 61,059 |

**B**

| | sequence reads | SL RNA tags | uniquely mapped iCLIP tags (except tRNAs/rRNAs) |
|---|---|---|---|
| **U1-70K_1** | 93,198 | 6,082 | 60,580 |
| **U1-70K_2** | 75,680 | 7,902 | 34,367 |

**U1C_1**

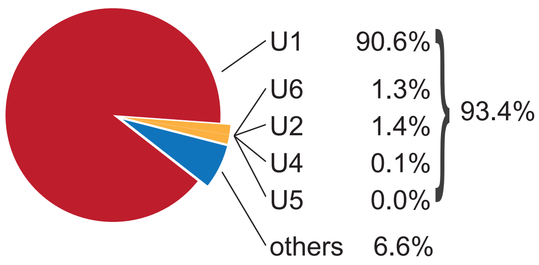


**U1-70K_1**

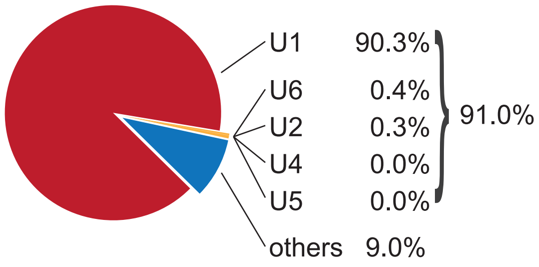


**U1C_2**

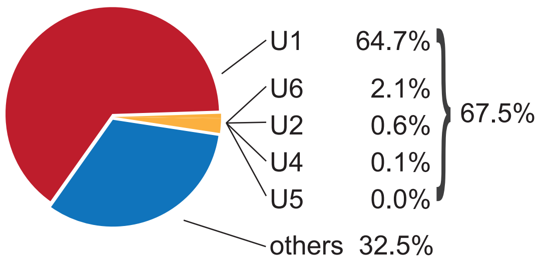


**U1-70K_2**

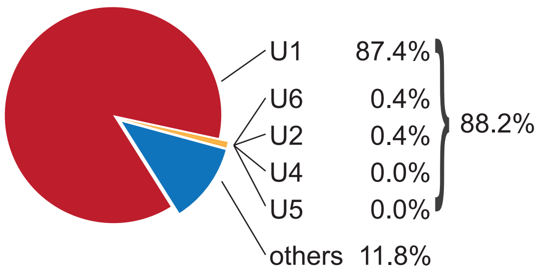


**U1C_3**

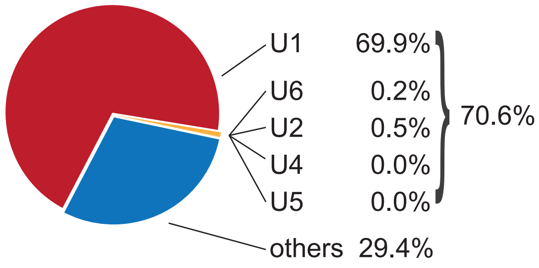

# Supplementary Figure S2

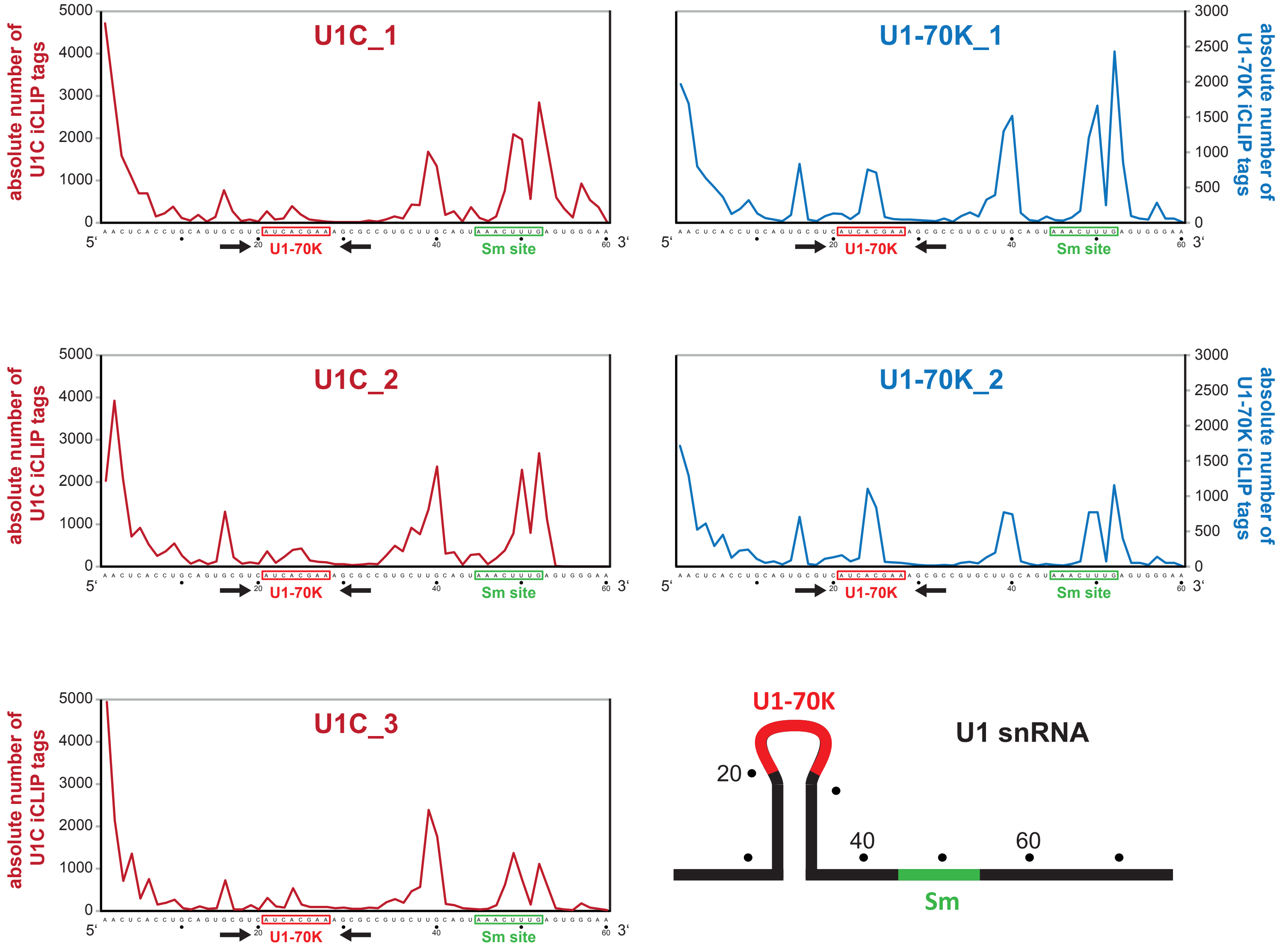

# Supplementary Figure S3

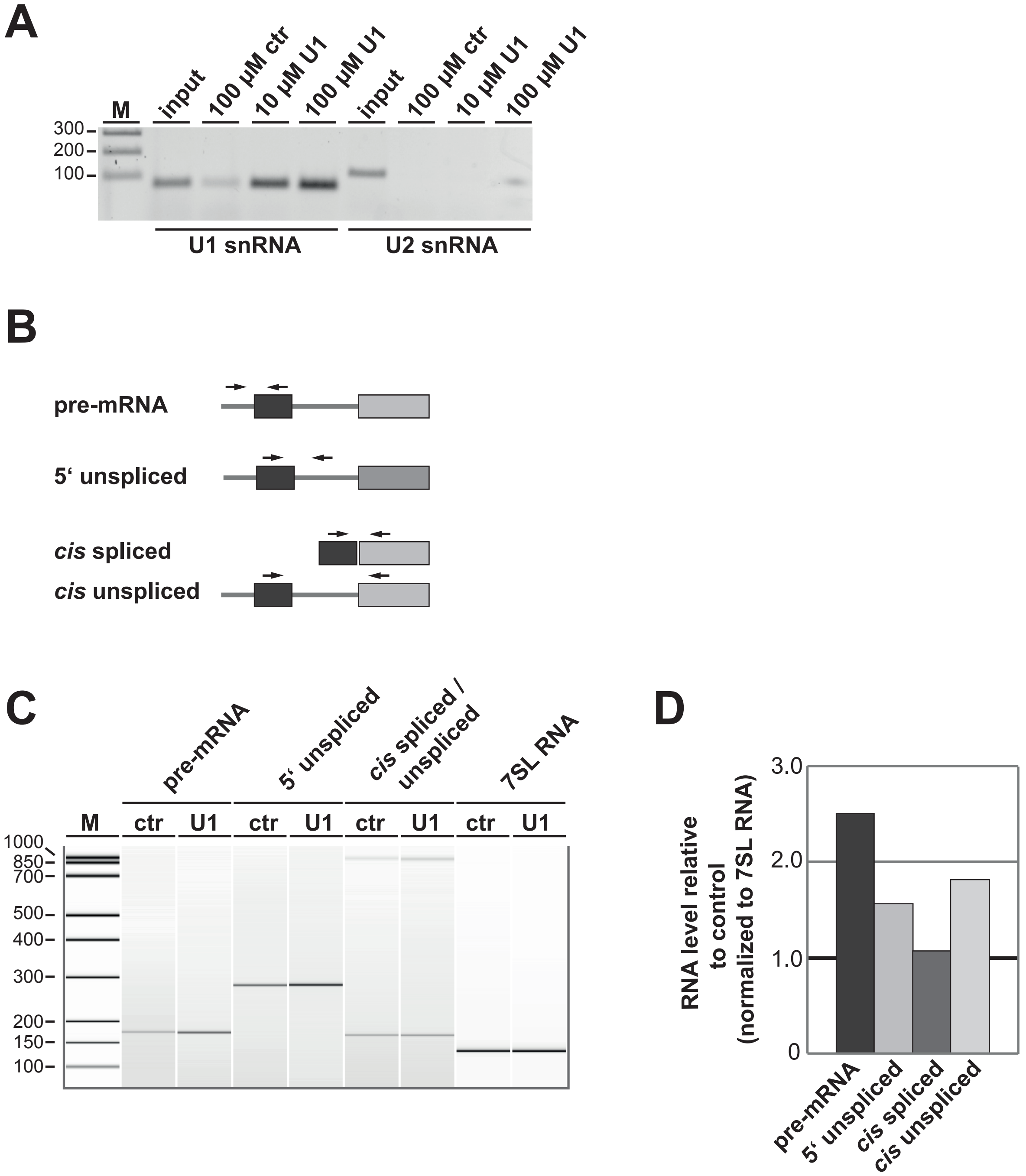

# Supplementary Figure S4

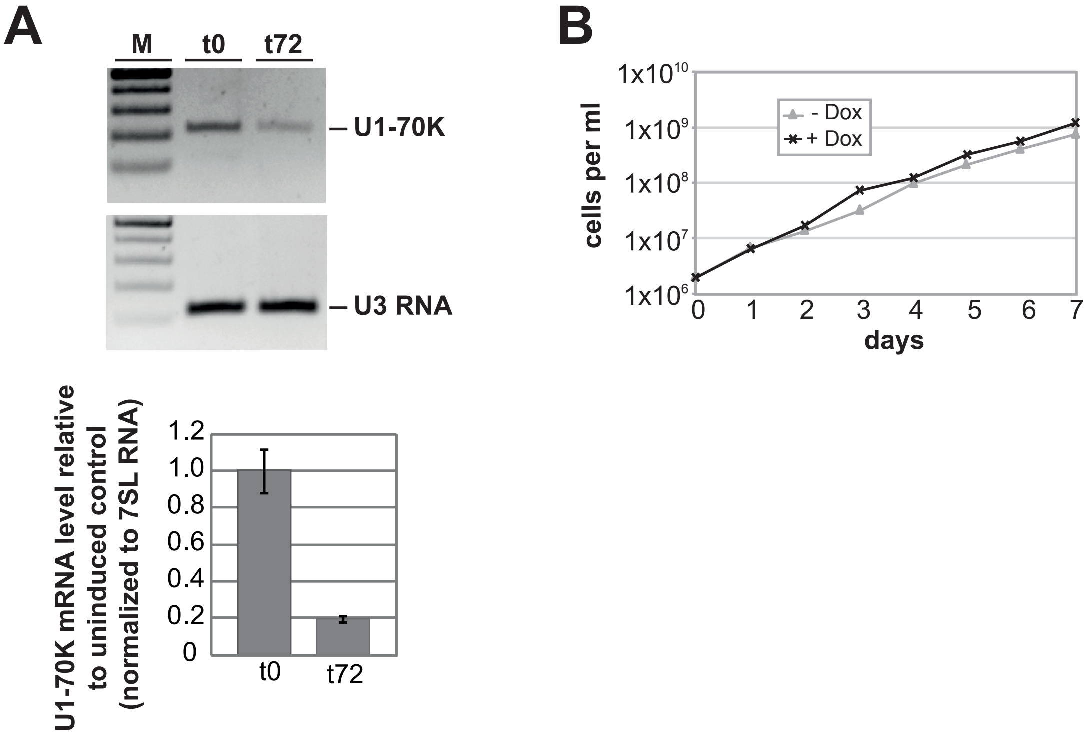

# Supplementary Figure S5

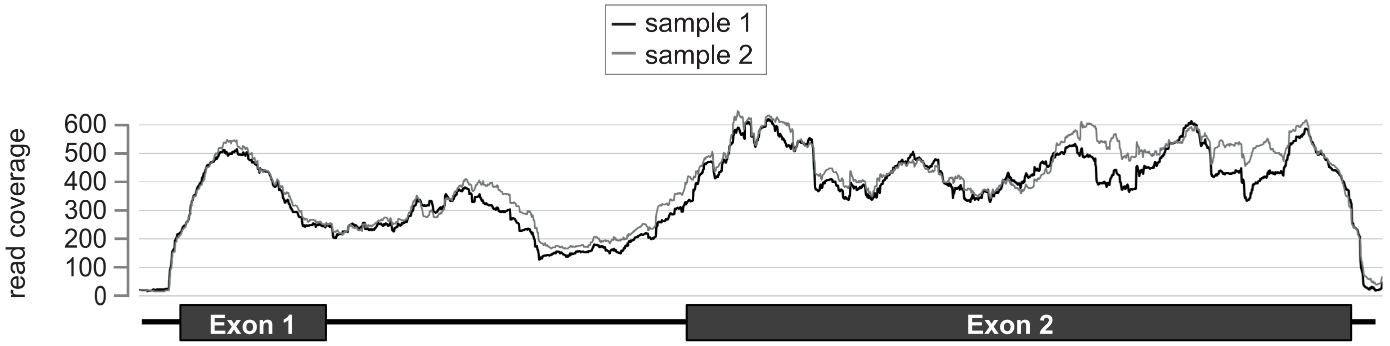


**Poly(A) polymerase**

# Supplementary Figure S6

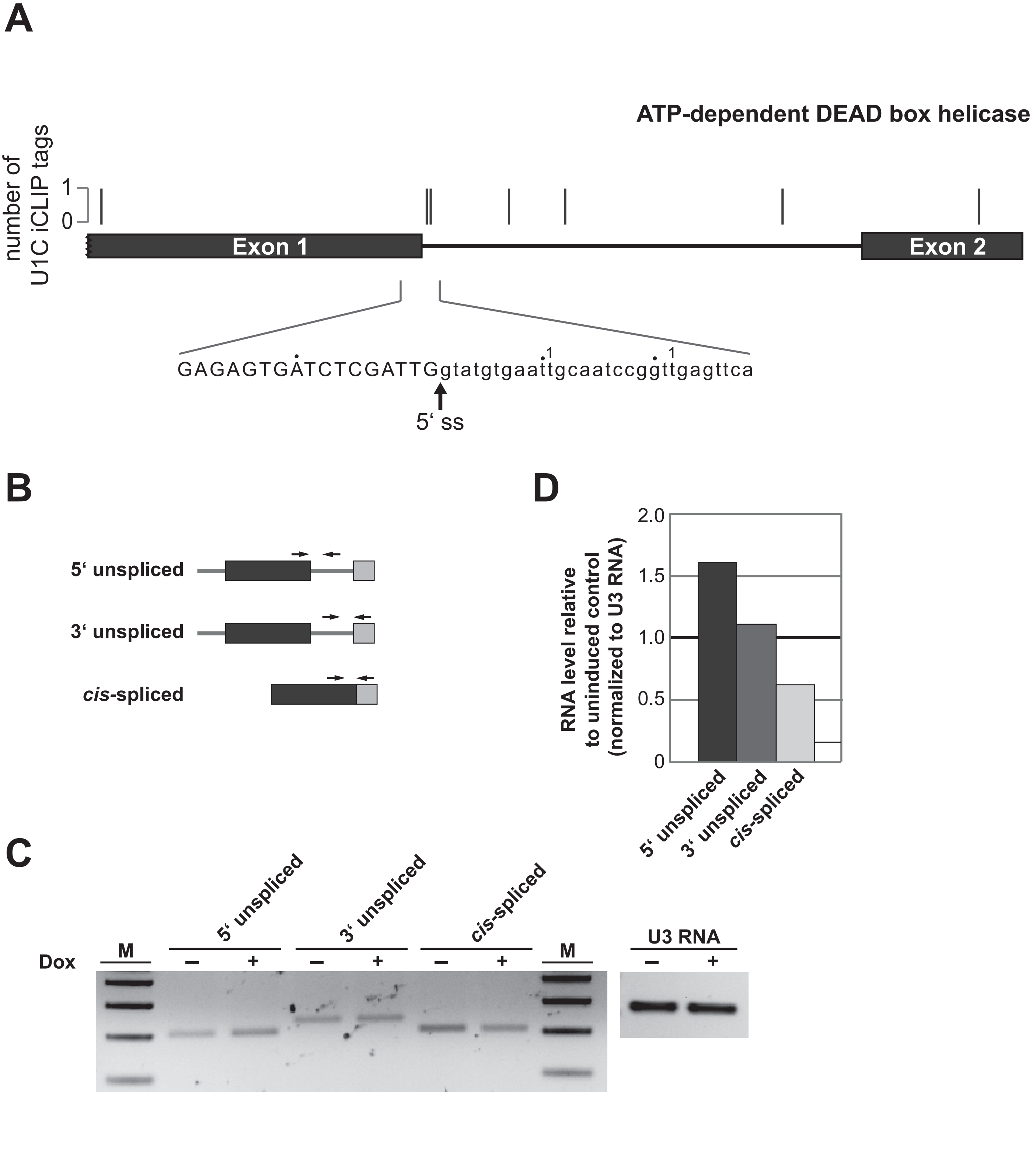

Supplement: SUPPLEMENTARY DATA [file supp_gku286_nar-00434-a-2014-File003.pdf]
